# Rapid Dot-Blot Immunoassay for Detecting Multiple *Salmonella enterica* Serotypes

**DOI:** 10.4014/jmb.2308.08006

**Published:** 2023-10-28

**Authors:** Jeongik Cho, Heymin Song, Hyun C. Yoon, Hyunjin Yoon

**Affiliations:** 1 Department of Molecular Science and Technology, Ajou University, Suwon 16499, Republic of Korea; 2 Department of Applied Chemistry and Biological Engineering, Ajou University, Suwon 16499, Republic of Korea

**Keywords:** *Salmonella*, serotype, dot-blot, detection

## Abstract

*Salmonella*, a major contributor to foodborne infections, typically causes self-limiting gastroenteritis. However, it is frequently invasive and disseminates across the intestinal epithelium, leading to deadly bacteremia. Although the genus is subdivided into >2,600 serotypes based on their antigenic determinants, only few serotypes are responsible for most human infections. In this study, a rapid dot-blot immunoassay was developed to diagnose multiple *Salmonella enterica* serotypes with high incidence rates in humans. The feasibility of 10 commercial antibodies (four polyclonal and six monoclonal antibodies) was tested using the 18 serotypes associated with 67.5% *Salmonella* infection cases in the United States of America (U.S.A) in 2016. Ab 3 (polyclonal; eight of 18 serotypes), Ab 8 (monoclonal; 13 of 18 serotypes), and Ab 9 (monoclonal; 10 of 18 serotypes) antibodies exhibited high detection rates in western blotting and combinations of two antibodies (Ab 3+8, Ab 3+9, and Ab 8+9) were applied to dot-blot assays. The combination of Ab 3+8 identified 15 of the tested 18 serotypes in 3 h, *i.e.*, *S*. Enteritidis, *S*. Typhimurium, *S*. Javiana, *S*. I 4,[5],12:i:-, *S*. Infantis, *S*. Montevideo, *S*. Braenderup, *S*. Thompson, *S*. Saintpaul, *S*. Heidelberg, *S*. Oranienburg, *S*. Bareilly, *S*. Berta, *S*. Agona, and *S*. Anatum, which were responsible for 53.7% *Salmonella* infections in the U.S. in 2016. This cost-effective and rapid method can be utilized as an on-site colorimetric method for *Salmonella* detection.

## Introduction

*Salmonella* infection, generally referred to as salmonellosis, is a gastrointestinal disorder caused by consuming *Salmonella*-contaminated foods [1]. *Salmonella* infection is typically linked to animal products such as poultry, egg, beef, and pork; however, other foods including fruits, vegetables, dried foods, infant formula, and pet foods may also be contaminated [2]. Based on the White–Kaufmann scheme, *Salmonella* is divided into two species, *Salmonella bongori* and *Salmonella enterica*, and *S*. *enterica* is further divided into six subspecies, *i.e.*, *S*. *enterica* subsp. *arizonae*, *S*. *enterica* subsp. *diarizonae*, *S*. *enterica* subsp. *enterica*, *S*. *enterica* subsp. *haoutenae*, *S*. *enterica* subsp. *indica*, and *S*. *enterica* subsp. *salamae* [3, 4]. Three surface structure determinants, including lipopolysaccharide (LPS), flagella, and capsular polysaccharide, differentiate them into approximately 2,659 serotypes [5, 6]. Among these, approximately 1,547 serotypes emanate from *S*. *enterica* subsp. *enterica* [6]. *Salmonella* serotypes are primarily transmitted via animal products, such as meats and eggs, can infect both humans and animals, and are generally known as nontyphoidal *Salmonella* (NTS) serotypes [7]. Common NTS infection symptoms include diarrhea, fever, abdominal cramps, and vomiting, and most healthy people recover within a week without hospitalization and medical treatment [8]. However, these NTS serotypes can be invasive (defined as invasive nontyphoidal *Salmonella*, iNTS) and cause fatal bacteremia and systemic infection, resulting in life-threatening complications [9].

According to the culture-confirmed human infection survey conducted by the Laboratory-based Enteric Disease Surveillance (LEDS) system, the 20 most prevalent serotypes in decreasing order were *Salmonella* Enteritidis, *Salmonella* Newport, *Salmonella* Typhimurium, *Salmonella* Javiana, *Salmonella* I 4,[5],12:i:-, *Salmonella* Infantis, *Salmonella* Muenchen, *Salmonella* Montevideo, *Salmonella* Braenderup, *Salmonella* Thompson, *Salmonella* Saintpaul, *Salmonella* Heidelberg, *Salmonella* Oranienburg, *Salmonella* Mississippi, *Salmonella* Typhi, *Salmonella* Bareilly, *Salmonella* Berta, *Salmonella* Agona, *Salmonella* Paratyphi B var., and *Salmonella* Anatum, which were associated with 69.2% incidence of culture-confirmed salmonellosis in the United States of America (U.S.) in 2016 [10]. Interestingly, 360 other serotypes were responsible for 16.5% salmonellosis incidence [10]. These 20 prevalent serotypes have been closely associated with human infection worldwide for the last decade, although the pecking orders varied with time and location [10]. We aimed to develop an on-site rapid diagnostic method to identify the dominant serotypes usually transmitted between humans and animal-based foods. The devised method targets 18 of the top 20 serotypes reported by LEDS system and excluded *S*. Typhi and *S*. Paratyphi because they do not colonize or inhabit animals as reservoir, except higher primates [5, 7].

From an epidemiological perspective, the surveillance of environmental serotypes is crucial for monitoring the emergence of pathogenic serotypes and preventing their transmission through the food chain [11, 12]. A dot-blot technique is a straightforward and rapid diagnostics where a small amount of bacterial lysate is directly dotted on a membrane and subsequently probed with a specific antibody to detect the target antigen [13]. Dot-blot assays are substantially more sensitive than enzyme-linked immunosorbent assay (ELISA) because nitrocellulose and polyvinylidene difluoride (PVDF) membranes have better protein- and LPS-binding capacities than the polystyrene plates used in ELISA [14]. Since this technique does not require highly skilled personnel or the expensive equipment needed for traditional immunology-based diagnosis, dot-blot immunoassays are affordable in most laboratories [15]. To exploit dot-blot immunoassay for a simple and reliable diagnosis for prevalent *Salmonella* serotypes, we determined a combination of antibodies to identify multiple dominant NTS serotypes and improved the method so that, excluding pre-cultivation time, the assay could be completed in 3 h.

## Materials and Methods

### Bacterial Strains and Growth Conditions

*Salmonella* strains used in this study are listed in Tables S1 and S2. Other bacterial strains used include *Escherichia coli* DH5α (ATCC 53868), *Vibrio cholerae* ATCC 14033, *Staphylococcus aureus* ATCC 29213, and *Bacillus cereus* ATCC 14579. Gram-negative bacteria, *i.e.*, *Salmonella*, *E. coli*, and *V. cholera* were cultivated in Luria–Bertani (LB) broth (Becton, Dickinson and Company, USA.) and gram-positive bacteria, *i.e.*, *S. aureus* and *B. cereus* were cultivated in brain–heart infusion (BHI) broth (Becton, Dickinson and Company). All bacterial strains were cultivated at 37°C.

### Antibodies

Ten different *Salmonella* antibodies were selected among commercially available antibodies and are listed in Table 1. The antibodies were produced in mouse or rabbit using diverse immunogens, including LPS (whole or partial), flagella, and whole cell preparation. Ab 3, Ab 5, Ab 6, and Ab 7 were rabbit polyclonal, whereas Ab 1, Ab 2, Ab 4, Ab 8, Ab 9, and Ab 10 were mouse monoclonal (Table 1). They were purchased from Abcam (USA.) and Invitrogen (Waltham, USA). The secondary antibodies used were goat anti-mouse IgG–HRP conjugate (#1706516; Bio-Rad Laboratories, Inc., USA), goat anti-rabbit IgG–HRP conjugate (#31460; Invitrogen), goat anti-mouse IgG–AP conjugate (A3562; Sigma-Aldrich, USA), and goat anti-rabbit IgG–alkaline phosphatase (A3687; Sigma-Aldrich).

### Western Blotting

Immunoblotting was conducted as described previously [16]. In brief, bacterial cells at the stationary growth phase were centrifuged at 12,000 ×*g* for 5 min, resuspended in 1× Laemmli sample buffer (Bio-Rad Laboratories, Inc.), and boiled for 5 min. The bacterial lysates were analyzed using 10% sodium dodecyl sulfate polyacrylamide electrophoresis (SDS-PAGE) gels and the protein fragments were transferred to PVDF membrane (Bio-Rad Laboratories, Inc.). Transfer buffer contained 25 mM Tris base, 0.2 M glycine, and 20% methanol (pH 8.5). The membrane was blocked with 5% skim milk solution (Tris Buffered Saline; TBS, 0.1% Tween-20, and 5% w/v skim milk) for 30 min. TBS contained 20 mM Tris-HCl and 150 mM NaCl. The membranes were then treated with primary antibodies (Table 1) at a dilution ratio of 1:20,000 and horseradish peroxidase (HRP)-conjugated secondary antibodies at a dilution ratio of 1:6,000 for 1 h, respectively. Antibody dilution buffer was TBS containing 0.1%Tween-20 and 5% skim milk and wash buffer (TBS-T) was TBS containing 0.1% Tween-20. All chemical reagents were purchased from Sigma-Aldrich, unless otherwise specified. As a size marker, Precision Plus Protein Dual Color Standards (#1610374; Bio-Rad Laboratories, Inc.) was used. Immunoblotting signals were developed using ECL Western Blotting Detection Reagents kit (GE Healthcare, USA.) and visualized using the ChemiDoc MP System (Bio-Rad Laboratories, Inc.).

### Dot-Blot Assay

**Fig. 1:**
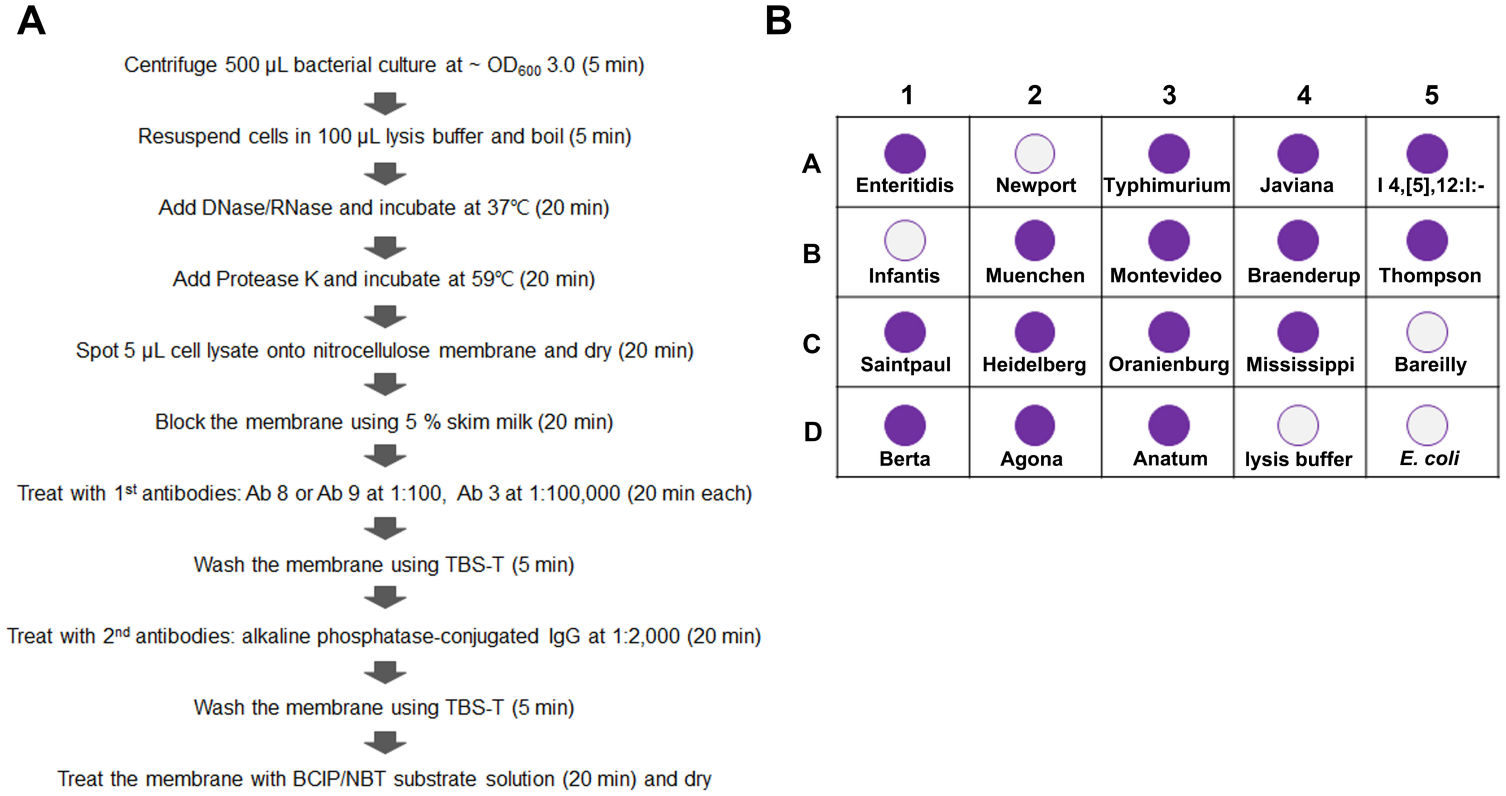
Scheme of the dot-blot immunoassay. (**A**) Flowchart of the designed method with the estimated time. (**B**) A diagram showing the designed locations of tested serotypes on a nitrocellulose membrane. White and purple circles are indicative of negative and positive interaction, respectively, with tested antibodies. Cell lysates of 18 *Salmonella* serotypes (A1– D3) used in optimizing the assay were spotted onto nitrocellulose membranes. Lysis buffer solution (D4) and *Escherichia coli* cell lysate (D5) were spotted as controls.

The experimental flowchart is shown in Fig. 1A. Bacterial cells were cultivated in LB broth until an OD_600_ of 3.0 (approximately 3 × 10^9^ CFU/ml) was reached and 500 μl cell suspension was centrifuged at 12,000 ×*g* for 5 min. The cells were resuspended in 100 μl lysis buffer (63 mM Tris-HCl, 0.1% β-mercaptoethanol, 2% SDS, and 10%glycerol) and boiled for 5 min. The cell lysate was treated with 10 μl DNase (Turbo DNA-free kit, #AM1907; Invitrogen) and RNase (#EN0531; Thermo Fisher Scientific, USA) at 37°C for 20 min and subsequently with 5 μl protease K (20 mg/ml, #AM2544; Invitrogen) at 59°C for 20 min. Then, 5 μl cell lysate solution was spotted on a nitrocellulose membrane (Bio-Rad Laboratories, Inc.) and dried for 20 min (Fig. 1B). The nitrocellulose membrane was blocked with 5% skim milk solution for 20 min and treated with primary antibodies (20 min for each antibody). Ab 8 and Ab 9 were diluted in 5% skim milk at a ratio of 1:100, whereas Ab 3 was diluted in 5% skim milk at a ratio of 1:100,000. When Ab 3 was applied together with Ab 8 or Ab 9, the membrane was first treated with Ab 8 or Ab9 (monoclonal antibody) before adding Ab 3 (polyclonal antibody). Antibody was diluted in TBS-T containing 5% skim milk. The membrane was washed using TBS-T buffer twice and treated with alkaline phosphatase-conjugated secondary antibodies diluted in 5% skim milk at a ratio of 1:2000 for 20 min. The membrane was washed using TBS-T buffer twice and treated with 20 mL substrate solution [0.4% nitrotetrazolium blue chloride (NBT, #N6876; Sigma-Aldrich) and 0.2% 5-bromo-4-chloro-3-indolyl phosphate disodium salt (BCIP, #B6149; Sigma-Aldrich) in a working buffer (0.1 M NaCl, 50 mM MgCl_2_, 0.1 M Tris-HCl)] for 20 min. The membrane was dried after purple spots developed.

## Results

### Assessment of the Cross-Reactivity of 10 Antibodies to Other Bacterial Pathogens

**Fig. 2:**
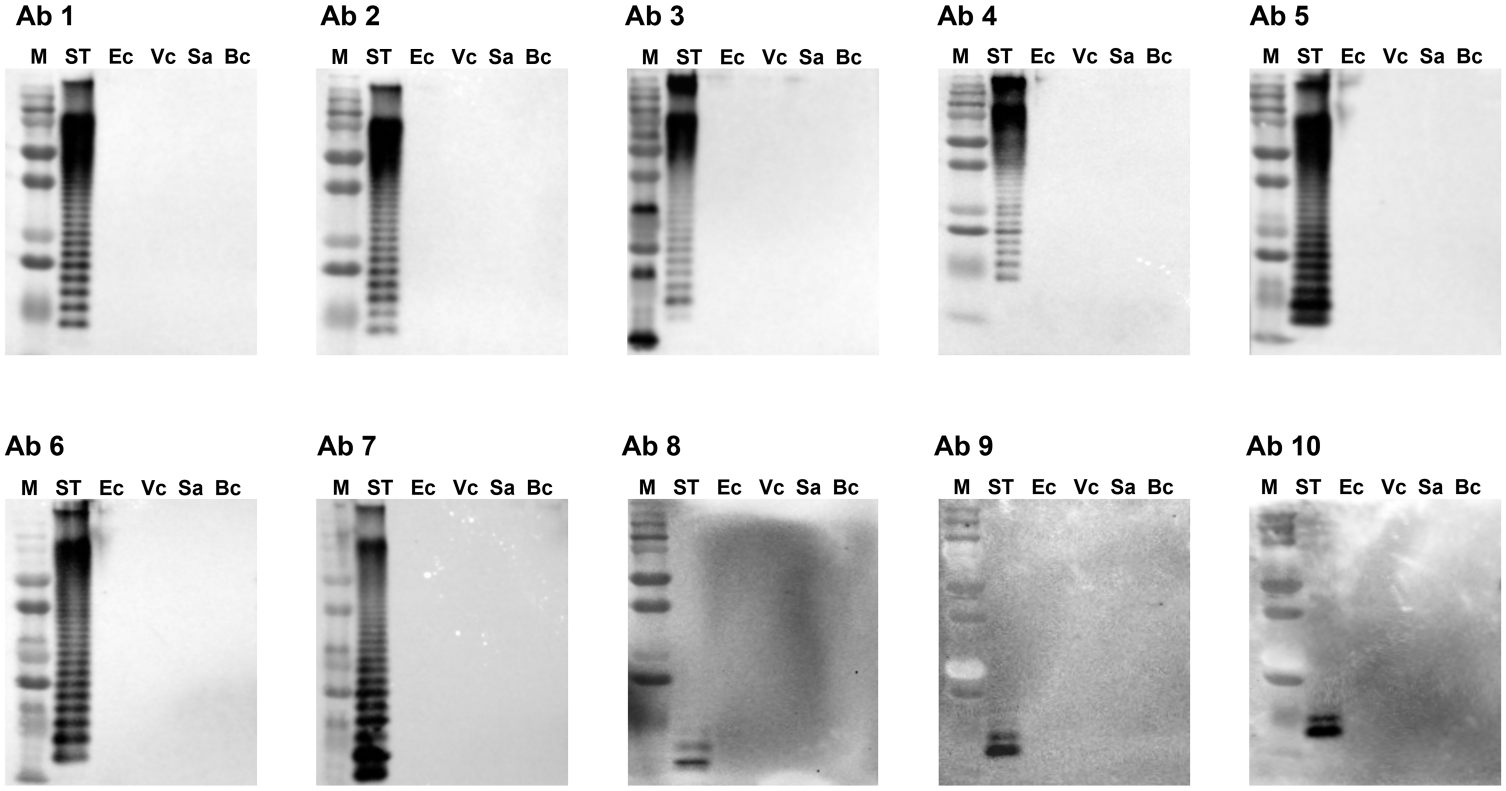
Cross-reactivity tests of 10 *Salmonella* antibodies (Ab 1–10) against five bacterial species in western blotting analyses. ST: *Salmonella* Typhimurium ATCC 14028; Ec: *Escherichia coli* ATCC 53868; Vc: *Vibrio cholerae* ATCC 14033; Sa: *Staphylococcus aureus* ATCC 29213; Bc: *Bacillus cereus* ATCC 14579. M: size marker.

The cross-reactivity of 10 different *Salmonella* antibodies (Table 1) was tested using five representative foodborne pathogens, including three gram-negative (*S*. Typhimurium, *E. coli*, and *V. cholera*) and two gram-positive (*S. aureus* and *B. cereus*) species (Fig. 2). The bacterial whole cell lysates were run on SDS-PAGE gels and probed with 10 different antibodies individually. All antibodies excluding Ab 8, Ab 9, and Ab 10 exhibited multiple ladder-like bands against *Salmonella*, while three antibodies of Ab 8, Ab 9, and Ab 10 against the common core oligosaccharide region of *Salmonella* LPS produced a few low-molecular weight bands with *Salmonella* cell lysates (Fig. 2). However, all antibodies were not cross-reactive with the other four bacterial species.

### Comparison of Antibody Selectivity against 18 *Salmonella* Serotypes

The detection effectiveness of the antibodies was examined using 18 *Salmonella* serotypes, *i.e.*, *S*. Enteritidis, *S*. Newport, *S*. Typhimurium, *S*. Javiana, *S*. I 4,[5],12:i:-, *S*. Infantis, *S*. Muenchen, *S*. Montevideo, *S*. Braenderup, *S*. Thompson, *S*. Saintpaul, *S*. Heidelberg, *S*. Oranienburg, *S*. Mississippi, *S*. Bareilly, *S*. Berta, *S*. Agona, and *S*. Anatum, which were responsible for 67.5% *Salmonella* infection in the U.S. in 2016 [10]. Incidence rates and tested strains of the 18 serotypes are listed in Table S1. The bacterial lysates were analyzed using SDS–PAGE and probed with 10 antibodies (Fig. S1). Ab 8, Ab 9, and Ab 10, which were generated using *Salmonella* core oligosaccharides as immunogens, displayed fewer bands with low-molecular weights than other antibodies. The selectivity of 10 antibodies against 18 serotypes are shown in Table 2. *S*. Typhimurium, *S*. Thompson, *S*. Saintpaul, and *S*. Heidelberg serotypes were detected by most tested antibodies. However, *S*. Newport was not detected by any antibody. Comparing the numbers of serotypes detected by each antibody indicated that Ab 8 had the highest detection rate (13/18) and detected *S*. Infantis, *S*. Montevideo, *S*. Mississippi, and *S*. Bareilly, which were not detected by the other antibodies (Fig. S1, Table 2). The detection rates of the four polyclonal antibodies were comparable (Ab 3: 8/18; Ab 5, Ab 6, and Ab 7: 7/18) and their selectivity spectra were also similar, overlapping *S*. Enteritidis, *S*. Typhimurium, *S*. Thompson, *S*. Saintpaul, *S*. Heidelberg, *S*. Berta, and *S*. Agona (Fig. S1, Table 2).

Owing to the selectivity and signal intensity (responsible for the sensitivity in dot-blot assays), Ab 3, Ab 8, and Ab 9 were selected for further dot-blot immunoassays. Ab 3 showed the highest detection rate among the four polyclonal antibodies and exhibited strong binding signals in the western blotting analysis. Ab 8 and Ab 9, despite having moderate signal intensities, demonstrated broad serotype spectra, covering 13 and 10 serotypes, respectively. The selectivity of three combinations (Ab 3+8, Ab 3+9, and Ab 8+9) was predicted based on the western blotting analyses (Table 2). Ab 3+8 and Ab 8+9 were estimated to detect 16 and 17 serotypes, respectively, which are responsible for 54.8% and 57.4% salmonellosis incidence, respectively, in the U.S. in 2016 [10]. Ab 3+9 was expected to detect 11 serotypes, which was inferior to the other two combinations.

### Application of a Combination of Two Antibodies to Dot-Blot Immunoassays

**Fig. 3:**
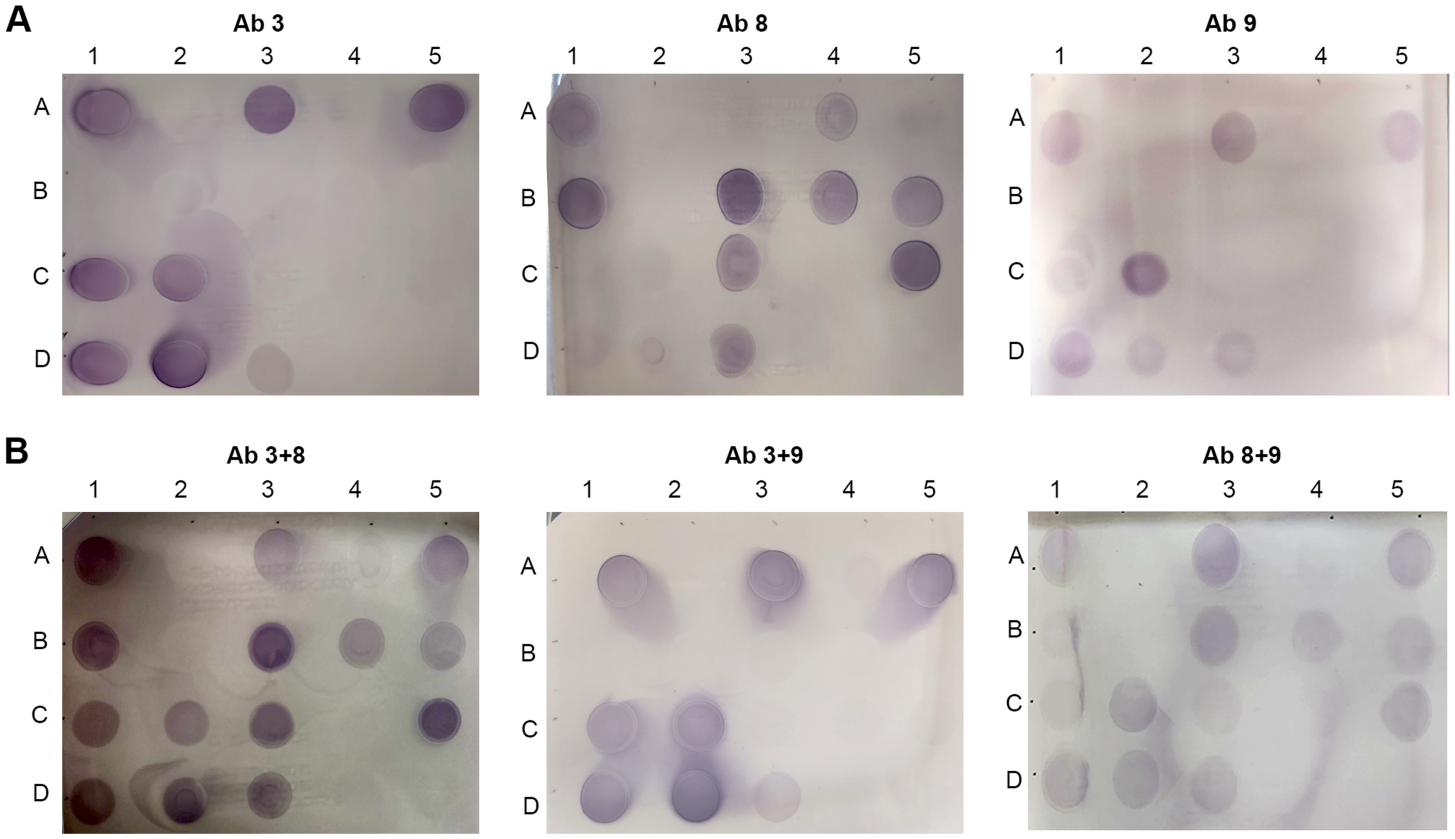
Dot-blot assays of three antibodies against 18 *Salmonella* serotypes. Cell lysates of 18 *Salmonella* serotypes were spotted as described in Fig. 1B and probed with Ab 3, Ab 8, and Ab 9, (**A**) individually or (**B**) in combination. As negative controls, lysis buffer solution (D4) and *Escherichia coli* cell lysate (D5) were tested in parallel.

Dot-blot assays were preformed using Ab 3, Ab 8, and Ab 9 individually (Fig. 3A) and in combination (Fig. 3B). The cell lysates of 18 *Salmonella* serotypes were dotted on nitrocellulose membranes, probed with primary and alkaline phosphatase-conjugated secondary antibodies, and treated with BCIP/NBT substrate solution as described in Materials and Methods (Fig. 1). Detection limit of three antibodies was estimated at approximately 10^6^ CFU in dot-blot assays using *S*. Typhimurium ATCC 19585 (Fig. S2). With regard to the differential antibody binding affinities between serotypes, *Salmonella* cells of approximately 7.5 × 10^7^ CFU were used for each tested serotype. The serotype selectivity of each antibody was compared between western blotting and dot-blot analyses in Table 3. Most serotypes detected in western blotting experiments were also detected in dot-blot assays with a few exceptions. Ab 3 failed to detect *S*. Braenderup and *S*. Thompson in dot-blot assays but detected *S*. I 4,[5],12:i:-and *S*. Anatum. Ab 8 did not detect *S*. Typhimurium and *S*. Mississippi in dot-blot analyses but detected *S*. Thompson. Ab 9 did not detect *S*. Javiana, *S*. Muenchen, and *S*. Thompson but detected *S*. I 4,[5],12:i:-.

Dot-blot assays were conducted using three combinations of two antibodies (Fig. 3B). Considering the weak binding signals of monoclonal antibodies, higher Ab 8 or Ab 9 concentrations were added before adding Ab 3 (Fig. 1). The optimized dot-blot assay using Ab 3+8 or Ab 8+9 identified 15 of 18 serotypes (Fig. 3B). The diagnosed 15 serotypes were overlapped between two combinations and were estimated to be associated with 53.7% salmonellosis cases in the U.S. in 2016 (Table 3). However, the detection sensitivity of Ab 8+9 was inferior to that of Ab 3+8, likely because of the weak signal of monoclonal antibodies.

**Fig. 4:**
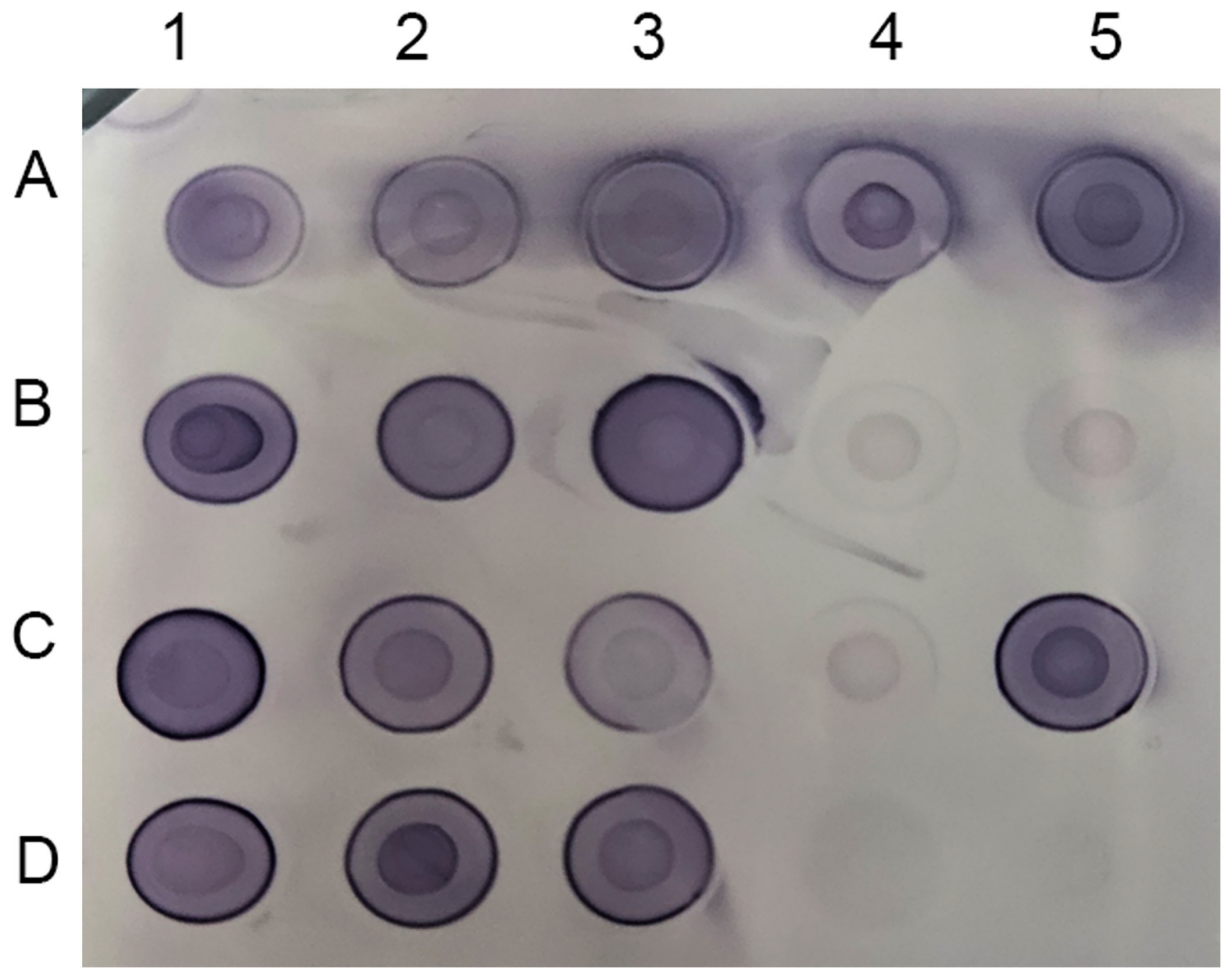
Diagnosis of 18 *Salmonella* strains using dot-blot immunoassay. Eighteen *Salmonella* strains belonging to seven different serotypes were subjected to the designed dot-blot immunoassay using Ab 3+8. A1: *S*. Enteritidis ATCC 4931; A2: *S*. Enteritidis FORC_019; A3: *S*. Enteritidis FORC_052; A4: *S*. Enteritidis ATCC 13076; A5: *S*. Enteritidis MFDS 1004839; B1: *S*. Typhimurium FORC_030; B2: *S*. Typhimurium NCCP 12219; B3: *S*. Typhimurium NCCP 14772; B4: *S*. Newport NCCP 12235; B5: *S*. Newport FORC_020; C1: *S*. Montevideo CCARM 8189; C2: *S*. Montevideo MFDS 1006814; C3: *S*. Infantis MFDS 1006818; C4: *S*. Muenchen KCPB 03; C5: *S*. Bareilly CCARM 8578; D1: *S*. Bareilly NCCP 11674; D2: *S*. Bareilly NCCP 16323; D3: *S*. Bareilly MFDS 1007637; D4: lysis buffer solution; D5: *Escherichia coli* ATCC 53868.

The designed dot-blot immunoassay using Ab 3+8 was applied to diagnose 18 *Salmonella* strains belonging to seven dominant serotypes (Fig. 4). Fifteen strains categorized into *S*. Enteritidis (five strains), *S*. Typhimurium (three strains), *S*. Montevideo (two strains), *S*. Infantis (one strain), and *S*. Bareilly (four strains) were successfully detected within 3 h. Noticeably, Ab 3+8 produced faint dot-blot signals against *S*. Newport (two strains) and *S*. Muenchen (one strain), which were not detectable in western blotting.

## Discussion

Traditional *Salmonella* detection methods are typically based on biological and physiological differences between *Salmonella* and the other bacterial species [17, 18]. Therefore, the procedures are labor-intensive and require at least several days [19]. Furthermore, traditional serotyping methods using antiserum agglutination with antigenic determinants require a comprehensive set of antisera and trained experts owing to the convoluted protocol, which hinders their extensive use as a popular diagnostic tool for general laboratories [19, 20]. With the developments in molecular biology, various rapid detection methods have been devised to supplement the drawbacks of conventional *Salmonella* detection techniques [19, 21]. Polymerase chain reaction (PCR)-based molecular diagnostic techniques are extremely quick and sensitive, but their extensive dissemination is constrained by the high cost of PCR operations [22]. Immunological diagnostic techniques, such as ELISA and immuno-chromatography, enable excellent *Salmonella* detection with high affinity [23-25]. However, these methods are time-consuming and yield low output. Moreover, the efficacy of antibodies is variable between serotypes and vulnerable to external environmental conditions [26, 27]. Besides, there are several other alternatives to the conventional methods, utilizing mass spectrometry, optical phenotyping, and electrochemical biosensors. Particularly, electrochemical biosensors combined with aptamer technology (such as aptasensors) exhibit excellent rapidity and sensitivity [28] and can be integrated into a portable biosensor device, which merits point-of-care testing [29, 30].

As an alternative immunoassay, dot-blot assays also utilize antibodies to detect target antigens but use nitrocellulose or PVDF membranes instead of polystyrene plates frequently used in ELISA. Antigens such as proteins and LPS bind to nitrocellulose and PVDF membranes more tightly than polystyrene plates [14, 15]. Therefore, dot-blot-based methods have considerably higher sensitivity than ELISA-based methods [14]. Dot-blot assay is a simplified method derived from western blotting. However, dot-blot assays do not require electrophoretic separation of antigen molecules on polyacrylamide gels and the procedures are substantially easier and faster than western blotting. Moreover, dot-blot assays are cost-effective. Besides, spotting bacterial lysates on membranes of randomized sizes enables screening large numbers of specimens for the presence of target antigens.

In contrast, dot-blot immunoassays possess a critical drawback owing to antibody specificity. Commercial antibodies against *Salmonella* generally recognize bacterial cell-surface epitopes present in the outer membrane protein, flagella, and LPS. The challenge is that even within the same species, structural components on the surface may vary between bacterial strains [31, 32]. Contrariwise, different bacterial species may have some similarity in their membranous structure. LPS molecules are complex glycolipids containing a hydrophobic moiety (lipid A), a core oligosaccharide, and a long-chain repeat-unit polysaccharide (O-antigen). *Salmonella* lipid A is a well-conserved archetypal structure comprising a bisphosphorylated glucosamine disaccharide carrying six acyl chains [33]. Although the core oligosaccharide architecture is also relatively conserved within a species, the O-antigen consists of variable oligosaccharide repeats, where the sugars and structural arrangements are different [34]. Owing to its hyper-variability in terms of length and sugars of repeat units, the O-antigen moiety provides various epitopes, determining the serological specificity between bacterial strains [35]. According to the Kauffman–White serological classification system, *Salmonella* has 46 O-serogroups [36]. Owing to the variable specificity and selectivity of antibodies between *Salmonella* serotypes, a single antibody may not detect every *Salmonella* serotype [37, 38]. Therefore, conventional dot-blot assays have been exploited to identify a specific *Salmonella* serotype such as *S*. Enteritidis. However, most monoclonal antibodies developed against *S*. Enteritidis had the potential to display cross-reactions with other *Salmonella* serotypes [15, 39, 40]. In the same context, the conventional *Salmonella* antibodies generated using LPS and/or flagella moieties as immunogens have the potential to interact with bacterial species with similar membranous structure. Especially, *E. coli* has many structural similarities to *Salmonella* spp. in LPS, flagella, and outer membrane proteins [41, 42]. Therefore, the reactivity of *Salmonella* antibodies should be interpreted carefully.

The dot-blot immunoassay developed in this study combined two different *Salmonella* antibodies with broad cross-reactivity against diverse *Salmonella* serotypes to diagnose multiple serotypes with high incidence rates in humans. The combination of Ab 3 and Ab 8 identified 15 predominant *Salmonella* serotypes (Fig. 3B; Table 3), which were responsible for 53.7% culture-confirmed salmonellosis cases in the U.S. in 2016 [10]. Notably, approximately 70% incidence of culture-confirmed salmonellosis was attributable to only 20 serotypes and the rest was ascribed to 360 serotypes (16.5%) and unknown agents (14.3%) [10]. The prevalent *Salmonella* serotypes were generally similar with minor changes for decades [10]. As a feasibility test, the developed dot-blot assay was applied to seven serotypes of the dominant 20 serotypes (Fig. 4): *S*. Enteritidis, *S*. Typhimurium, *S*. Newport, *S*. Infantis, *S*. Muenchen, *S*. Montevideo, and *S*. Bareilly. Three serotypes of *S*. Enteritidis, *S*. Typhimurium, and *S*. Newport are typically recognized as the top 3 serotypes with the highest incidence rates [10]. Salmonellosis attributable to the other 4 serotypes is growing recently, although the case numbers are much lower than those by the top 3 serotypes [43-46]. Fifteen strains belonging to *S*. Enteritidis, *S*. Typhimurium, *S*. Montevideo, *S*. Infantis, and *S*. Bareilly were successfully detected, whereas 3 strains of *S*. Newport and *S*. Muenchen produced weak dot-blot signals. Interestingly, Ab 3 and Ab 8 failed to detect *S*. Newport and *S*. Muenchen in western blotting (Fig. S1, Table 2). In comparison with PVDF membranes, nitrocellulose membranes used in the dot-blot assay might exert better binding affinity with various antigenic materials including oligosaccharides, lipids, and proteins, as observed previously [47, 48]. Varied attempts, such as pretreating membranes to increase hydrophobic interactions, adding more antibodies, and using more sensitive reporter enzymes, could be carried out to improve the detection sensitivity.

The drawback of conventional *Salmonella* dot-blot immunoassays, which is attributable to the cross-reactivity of most commercial antibodies across diverse *Salmonella* serotypes, was exploited as a counterplot to diagnose multiple serotypes using two antibodies in this study. Combination of polyclonal and monoclonal antibodies broadened the detection spectrum, covering 15 serotypes, and intensified the dot-blot signals. Combination with different and more antibodies can extend the diagnostic spectrum. Considering the simplicity and rapidity, the devised dot-blot immunoassay can be employed for an on-site rapid diagnosis of harmful *Salmonella* serotypes in the food industry.

## Supplemental Materials

Supplementary data for this paper are available on-line only at http://jmb.or.kr.



## Figures and Tables

**Table 1:** Ten primary antibodies used in this study.

ID	Product	Immunogen	Host	Isotype
Ab 1	*Salmonella* Typhimurium LPS antibody [1E6] (ab8274)	*S*. Typhimurium LPS	Mouse (mAb)	IgG1
Ab 2	*Salmonella* LPS antibody [se-01] (ab243104)	*S*. Typhimurium LPS	Mouse (mAb)	IgG1
Ab 3	*Salmonella* polyclonal antibody (PA1-7244)	*Salmonella* O & H antigens	Rabbit (pAb)	IgG
Ab 4	*Salmonella* monoclonal antibody [F68C] (MA1-7443)	*Salmonella* sp. preparation	Mouse (mAb)	IgG1
Ab 5	*Salmonella* antibody (ab35156)	Mixture of *S*. Enteritidis, *S*. Typhimurium and *S*. Heidelberg	Rabbit (pAb)	IgG
Ab 6	*Salmonella* antibody (ab252742)	Mixture of *S*. Enteritidis, *S*. Typhimurium and *S*. Heidelberg	Rabbit (pAb)	IgG
Ab 7	*Salmonella* polyclonal antibody (PA1-20811)	Mixture of *S*. Enteritidis, *S*. Typhimurium and *S*. Heidelberg	Rabbit (pAb)	IgG
Ab 8	*Salmonella* LPS monoclonal antibody [F62C] (MA1-7447)	*Salmonella* sp. common core	Mouse (mAb)	IgG2b
Ab 9	*Salmonella* LPS monoclonal antibody [D46J] (MA5-18257)	*Salmonella* sp. common core	Mouse (mAb)	IgG2a
Ab 10	*Salmonella* LPS monoclonal antibody [A99H] (MA5-18258)	*Salmonella* sp. common core	Mouse (mAb)	IgG2a

**Table 2:** Spectrum of 10 *Salmonella* antibodies against 18 serotypes in western blotting.

	Antibody (Ab No.)												
	1	2	3	4	5	6	7	8	9	10	3+8	3+9	8+9
*S*. Enteritidis	−^3^	+	+ +	−	+ +	+ +	+ +	+	+ +	+	+ +	+ +	+ +
*S*. Newport	−	−	−	−	−	−	−	−	−	−	−	−	−
*S*. Typhimurium	+ + +	+ + +	+ + +	+ + +	+ + +	+ + +	+ + +	+	+ +	+ +	+ + +	+ + +	+ +
*S*. Javiana	−	−	−	−	−	−	−	+	+	+ + +	+	+	+
*S*. I 4,[5],12:i:-	−	−	−	−	−	−	−	+	−	+ + +	+	−	+
*S*. Infantis	−	−	−	−	−	−	−	+ +	−	−	+ +	−	+ +
*S*. Muenchen	−	−	−	−	−	−	−	−	+	−	−	+	+
*S*. Montevideo	−	−	−	−	−	−	−	+ +	−	−	+ +	−	+ +
*S*. Braenderup	−	−	−	−	−	−	−	+ +	−	−	+ +	+	+ +
*S*. Thompson	+ + +	+ + +	+ + +	+ +	+ + +	+ + +	+ + +	−	+	+	+ + +	+ + +	+
*S*. Saintpaul	+ + +	+ + +	+ + +	+ + +	+ + +	+ + +	+ + +	−	+ +	-	+ + +	+ + +	+ +
*S*. Heidelberg	+ + +	+ + +	+ + +	+ + +	+ + +	+ + +	+ + +	−	+ +	+	+ + +	+ + +	+ +
*S*. Oranienburg	−	−	−	−	−	−	−	+ +	−	+	+ +	−	+ +
*S*. Mississippi	−	−	−	−	−	−	−	+	−	−	+	−	+
*S*. Bareilly	−	−	−	−	−	−	−	+ +	−	−	+ +	−	+ +
*S*. Berta	−	−	+ + +	−	+ + +	+ + +	+ + +	+	+ +	+	+ + +	+ + +	+ +
*S*. Agona	−	+ + +	+ + +	−	+ + +	+ + +	+ + +	+	+ +	−	+ + +	+ + +	+ +
*S*. Anatum	−	−	−	−	−	−	−	+ +	+	+	+ +	+	+ +
Detection rate^1^	4/18	6/18	7/18	5/18	7/18	7/18	7/18	13/18	10/18	9/18	16/18	11/18	17/18
Incidence (%)^2^	14.8	32.4	35.3	16.9	33.2	33.2	33.2	49.8	42.2	43.3	54.8	44.3	57.4

^1^Detection rate: Number of detected serotypes out of tested 18 serotypes.

^2^Incidence (%): Percentage of salmonellosis incidence associated with the detectable serotypes. Incidence was retrieved from the culture-confirmed human infection survey by LEDS. https://www.cdc.gov/nationalsurveillance/pdfs/2016-Salmonella-report-508.pdf.

^3^Symbols of +/-: Detected/not detected. More symbols indicate stronger detection signals.

**Table 3:** Spectrum of three *Salmonella* antibodies against 18 serotypes in dot-blot assay.

	Antibody (Ab No.)								
	3		8		9		3+8	3+9	8+9
	WB^1^	DB^2^	WB	DB	WB	DB	DB	DB	DB
*S*. Enteritidis	+ +^5^	+ +	+	+	+ +	+	+ +	+ +	+
*S*. Newport	−	−	−	−	−	−	−	−	−
*S*. Typhimurium	+ + +	+ +	+	−	+ +	+	+ +	+ +	+
*S*. Javiana	−	−	+	+	+	−	+	−	+
*S*. I 4,[5],12:i:-	−	+ +	+	+	−	+	+ +	+ +	+
*S*. Infantis	−	−	+ +	+ +	−	−	+ +	−	+
*S*. Muenchen	−	−	−	−	+	−	−	−	-
*S*. Montevideo	−	−	+ +	+ +	-	−	+ +	−	+
*S*. Braenderup	+	−	+ +	+	−	−	+	−	+
*S*. Thompson	+ + +	−	−	+	+	−	+	−	+
*S*. Saintpaul	+ + +	+ +	−	−	+ +	+	+ +	+ +	+
*S*. Heidelberg	+ + +	+ +	−	−	+ +	+	+ +	+ +	+
*S*. Oranienburg	−	−	+ +	+	−	−	+ +	−	+
*S*. Mississippi	−	−	+	−	−	−	−	−	−
*S*. Bareilly	−	−	+ +	+ +	−	−	+ +	−	+
*S*. Berta	+ + +	+ +	+	+	+ +	+	+ +	+ +	+
*S*. Agona	+ + +	+ +	+	+	+ +	+	+ +	+ +	+
*S*. Anatum	−	+	+ +	+	+	+	+ +	+	+
Detection rate^3^	8/18	8/18	13/18	12/18	10/18	8/18	15/18	8/18	15/18
Incidence (%)^4^	35.3	36.8	49.8	40.6	42.2	36.8	53.7	36.8	53.7

^1^WB: western blotting analysis.

^2^DB: Dot-blot immunoassay.

^3^Detection rate: Number of detected serotypes out of tested 18 serotypes.

^4^Incidence (%): Percentage of salmonellosis incidence associated with the detectable serotypes. Incidence was retrieved from the culture-confirmed human infection survey by LEDS. https://www.cdc.gov/nationalsurveillance/pdfs/2016-Salmonella-report-508.pdf.

^5^Symbols of +/-: Detected/not detected. More symbols indicate stronger detection signals.
