# Supplementary material for: Rapid Dot-Blot Immunoassay for Detecting Multiple Salmonella enterica Serotypes
Source: J Microbiol Biotechnol. 2023 Oct 28;34(2):340–8. doi: 10.4014/jmb.2308.08006 (PMC10940738; doi:10.4014/jmb.2308.08006)
Supplement: Supplementary file 1 [file jmb-34-2-340-supple.pdf]

J. Microbiol. Biotechnol.
https://doi.org/10.4014/jmb.2308.08006

Supplementary Tables and Figures

Table S1. *Salmonella* serotypes used in designing dot-blot immunoassay.

Dot-blot ID[1]	Serotype	Reported No.[2]	Incidence[3] (%)	Strain	Reference
A1	*S.* Enteritidis	7,830	2.44 (16.8)	MFDS 1010897	MFDS[4]
A2	*S.* Newport	4,728	1.47 (10.1)	MFDS 1005422	MFDS
A3	*S.* Typhimurium	4,581	1.43 (9.8)	ATCC 19585	ATCC[5]
A4	*S.* Javiana	2,719	0.85 (5.8)	2080H	FDA[6]
A5	*S.* I 4,[5],12:I:-	2,179	0.68 (4.7)	MFDS 1004858	MFDS
B1	*S.* Infantis	1,281	0.4 (2.7)	S22	KCPB[7]
B2	*S.* Muenchen	1,216	0.38 (2.6)	S06	KCPB
B3	*S.* Montevideo	1,018	0.32 (2.2)	S04	KCPB
B4	*S.* Braenderup	1,001	0.31 (2.1)	MFDS 1008393	MFDS
B5	*S.* Thompson	792	0.25 (1.7)	CCARM 8530	CCARM[8]
C1	*S.* Saintpaul	778	0.24 (1.7)	CCARM 8581	CCARM
C2	*S.* Heidelberg	754	0.23 (1.6)	3390H	FDA
C3	*S.* Oranienburg	692	0.22 (1.5)	1410H	FDA
C4	*S.* Mississippi	536	0.17 (1.1)	2883H	FDA
C5	*S.* Bareilly	412	0.13 (0.9)	1955H	FDA
D1	*S.* Berta	369	0.11 (0.8)	BA0000581	KVCC[9]
D2	*S.* Agona	362	0.11 (0.8)	S12	KCPB
D3	*S.* Anatum	257	0.08 (0.6)	1904H	FDA
Subtotal (serotyped)		32,271	9.82 (67.5)		
Subtotal (others and unknown)		15,118	4.69 (32.5)		
Total		46,623	14.51 (100)		

[1]Dot-blot ID: ID used in Fig. 1 and Fig. 3.

[2]Reported No.: Culture-confirmed human *Salmonella* infections reported in US 2016.
https://www.cdc.gov/nationalsurveillance/pdfs/2016-Salmonella-report-508.pdf

[3]Incidence: Culture-confirmed human salmonellosis incidence per 100,000.
https://www.cdc.gov/nationalsurveillance/pdfs/2016-Salmonella-report-508.pdf

[4]MFDS: Ministry of Food and Drug Safety, Korea

[5]ATCC: American Type Culture Collection

[6]FDA: US Food and Drug Administration

[7]KCPB: Korea Consumer Protection Board

[8]CCARM: Culture Collection of Antimicrobial Resistance Microbes, Korea

[9]KVCC: Korea Veterinary Culture Collection

Table S2. *Salmonella* serotypes tested in the developed dot-blot immunoassay.

Dot-blot ID[1]	Serotype	Strain	Reference
A1	*S.* Enteritidis	ATCC 4931	ATCC[2]
A2	*S.* Enteritidis	FORC_019	FORC[3]
A3	*S.* Enteritidis	FORC_052	FORC
A4	*S.* Enteritidis	ATCC 13076	ATCC
A5	*S.* Enteritidis	MFDS 1004839	MFDS[4]
B1	*S.* Typhimurium	FORC_030	FORC
B2	*S.* Typhimurium	NCCP 12219	NCCP[5]
B3	*S.* Typhimurium	NCCP 14772	NCCP
B4	*S.* Newport	NCCP 12235	NCCP
B5	*S.* Newport	FORC_020	FORC
C1	*S.* Montevideo	CCARM 8189	CCARM[6]
C2	*S.* Montevideo	MFDS 1006814	MFDS
C3	*S.* Infantis	MFDS 1006818	MFDS
C4	*S.* Muenchen	KCPB 03	KCPB[7]
C5	*S.* Bareilly	CCARM 8578	CCARM
D1	*S.* Bareilly	NCCP 11674	NCCP
D2	*S.* Bareilly	NCCP 16323	NCCP
D3	*S.* Bareilly	MFDS 1007637	MFDS

[1]Dot-blot ID: ID used in Fig. 4.

[2]ATCC: American Type Culture Collection

[3]FORC: Food-borne pathogen Omics Research Center, Korea

[4]MFDS: Ministry of Food and Drug Safety, Korea

[5]NCCP: National Culture Collection for Pathogens, Korea

[6]CCARM: Culture Collection of Antimicrobial Resistance Microbes, Korea

[7]KCPB: Korea Consumer Protection Board

Figure S1.

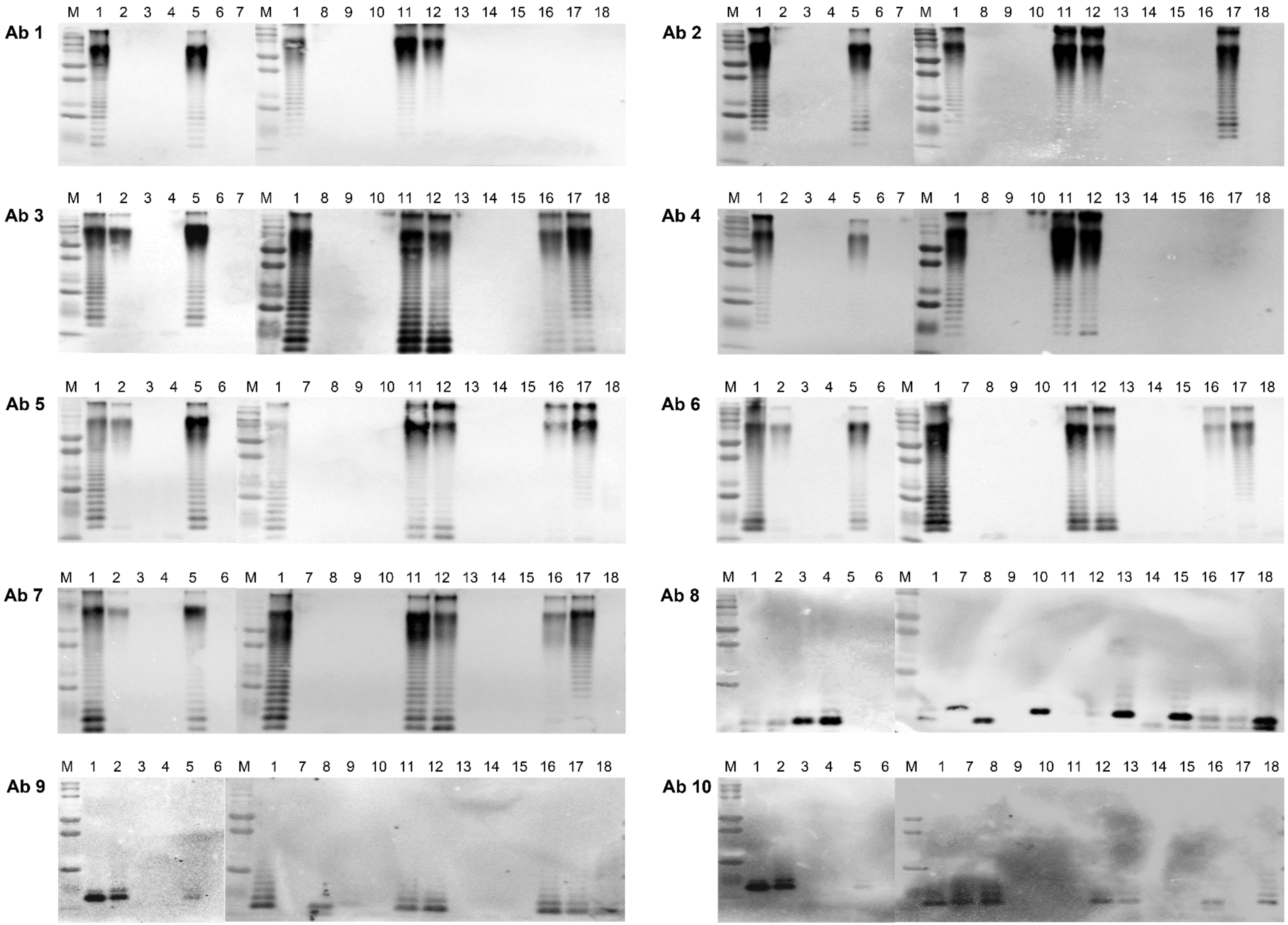
Ab 1
M 1 2 3 4 5 6 7 M 1 8 9 10 11 12 13 14 15 16 17 18
Ab 2
M 1 2 3 4 5 6 7 M 1 8 9 10 11 12 13 14 15 16 17 18
Ab 3
M 1 2 3 4 5 6 7 M 1 8 9 10 11 12 13 14 15 16 17 18
Ab 4
M 1 2 3 4 5 6 7 M 1 8 9 10 11 12 13 14 15 16 17 18
Ab 5
M 1 2 3 4 5 6 M 1 7 8 9 10 11 12 13 14 15 16 17 18
Ab 6
M 1 2 3 4 5 6 M 1 7 8 9 10 11 12 13 14 15 16 17 18
Ab 7
M 1 2 3 4 5 6 M 1 7 8 9 10 11 12 13 14 15 16 17 18
Ab 8
M 1 2 3 4 5 6 M 1 7 8 9 10 11 12 13 14 15 16 17 18
Ab 9
M 1 2 3 4 5 6 M 1 7 8 9 10 11 12 13 14 15 16 17 18
Ab 10
M 1 2 3 4 5 6 M 1 7 8 9 10 11 12 13 14 15 16 17 18

Fig. S1. Spectra of 10 antibodies against 18 *Salmonella* serotypes. *Salmonella* cell lysates were probed with 10 different *Salmonella* antibodies from Ab 1 to Ab 10 in western blotting analyses. 1: *S*. Typhimurium ATCC 19585; 2: *S*. Enteritidis MFDS 1010897; 3: *S.* Infantis S22; 4: *S.* Montevideo S04; 5: *S.* Thompson CCARM 8530; 6: *S.* Newport MFDS 1005422; 7: *S.* I 4,[5],12:I:- MFDS 1004858; 8: *S.* Javiana 2080H; 9: *S.* Muenchen S06; 10: *S.* Braenderup MFDS 1008393; 11: *S.* Saintpaul CCARM 8581; 12: *S.* Heidelberg 3390H; 13: *S.* Oranienburg 1410H; 14: *S.* Mississippi 2883H; 15: *S.* Bareilly 1955H; 16: *S.* Berta BA0000581; 17: *S.* Agona S12; *S.* Anatum 1904H. M indicates size marker.

Figure S2

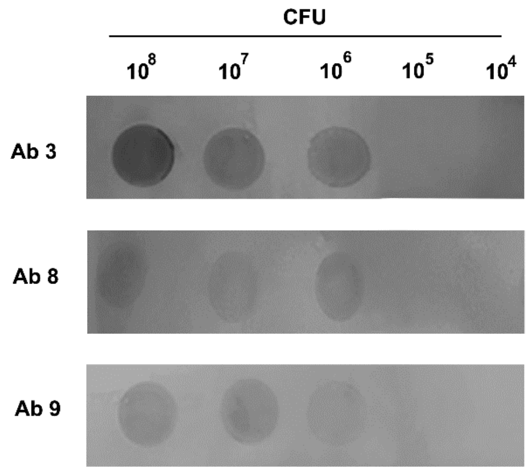

Fig. S2. Detection limit estimation of Ab 3, Ab 8, and Ab 9 in dot-blot assays. *Salmonella* Typhimurium ATCC 19585 cells from 10^4 CFU to 10^8 CFU were treated with three antibodies individually in dot-blot assays. Ab 3 was diluted at 1:100,000 and Ab 8 and Ab 9 were diluted at 1: 100.
